# Extreme‐Thickness Meta‐Membrane for Controlling Terahertz Vectorial Beams

**DOI:** 10.1002/advs.75898

**Published:** 2026-05-29

**Authors:** Yufei Song, Yixiang Xu, Yikai Xu, Yuanxi Liu, Shaojie Ma, Qiong He, Zhuo Wang, Lei Zhou

**Affiliations:** ^1^ State Key Laboratory of Surface Physics Key Laboratory of Micro and Nano Photonic Structures (Ministry of Education) and Department of Physics Fudan University Shanghai China; ^2^ College of Future Information Technology Fudan University Shanghai China; ^3^ Shanghai Key Laboratory of Metasurfaces for Light Manipulation Fudan University Shanghai China

**Keywords:** extreme‐thickness structures, metasurfaces, near‐field coupling, terahertz, vectorial bessel beams

## Abstract

Manipulating terahertz (THz) beams at will in transmission mode is desired, but conventional devices are bulky and unfavourable for optical integration. Metasurfaces (MSs) offer a versatile platform for light manipulation, but transmission‐mode MSs need sufficient thickness to achieve wide‐range phase modulation. This work proposes a new type of flexible MSs, consisting of two metallic screens with air slits exhibiting tailored geometries and orientations, to manipulate the wave‐fronts and polarization properties of THz beams in transmission mode. In contrast to conventional Huygens MSs involving impedance‐matched electric and magnetic resonances, our meta‐system relies on near‐field coupling of two slit resonances and thus can be extremely thin (∼λ/30). This work employs leaky eigenmode theory to analyse the transmission properties of arrays of coupled air slits and experimentally demonstrates that the phase and polarization of the transmitted THz wave can be modulated by varying the structures and orientation angles of two air slits. Using these coupled air‐slits as meta‐atoms, three THz MSs are constructed to generate scalar and radially polarized vectorial Bessel beams under 0.39 THz illumination. The results are confirmed by full‐wave simulations. This approach enables transmissive THz metadevices for applications such as biosensing, communications, and displays.

## Graphical abstract

We propose ultra‐thin (λ/30) transmissive terahertz metasurfaces combining near‐field resonant and detour phases in coupled‐slit meta‐atoms, enabling continuous phase and polarization control beyond conventional designs. Based on leaky‐mode theory, scalar and vectorial terahertz Bessel beams at 0.39 THz are experimentally demonstrated, with measurements agreeing well with full‐wave simulations.

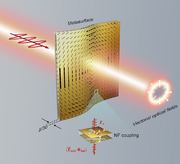

## 1 Introduction

Terahertz (THz) waves exhibit great application potentials in information technology, owing to their strong penetrability and unique spectral fingerprint characteristics [1, 2, 3, 4, 5, 6, 7]. In particular, vectorial THz beams, possessing inhomogeneous polarization distributions on their wave‐fronts, can carry/detect more information than their scalar counterparts, thus attracting intensive attention recently [8, 9, 10, 11, 12]. However, traditional systems to generate and control vectorial THz beams consist of a series of discrete optical components (e.g., polarizers, spiral phase plates, etc.), which are bulky and complex being unfavorable for optical integration [13, 14, 15, 16]. In fact, conventional optical devices rely on propagation phases of light passing across them to manipulate the properties of transmitted beams, which are inevitably thick (compared to the wavelength) and exhibit limited functionalities.

Metasurfaces (MSs), extreme‐thickness metamaterials composed of deep‐subwavelength microstructures (e.g., meta‐atoms) possessing tailored electromagnetic (EM) responses arranged in certain pre‐determined sequences, have exhibited powerful capabilities to control EM waves based on Huygens’ principle [17, 18, 19, 20, 21, 22]. Through carefully engineering the phase profiles of MSs, many fascinating wave‐manipulation effects have been demonstrated, including anomalous refraction/reflection [23, 24, 25, 26, 27], meta‐lens imaging [28, 29, 30, 31, 32], meta‐holography [33, 34, 35, 36, 37], etc. Later, employing meta‐atoms that can control both phases and polarizations of locally scattered waves, a series of MSs were realized to generate vectorial beams at different frequencies [38, 39]. However, most of these metadevices are working in reflection mode, which are inconvenient in realistic applications. To construct a high‐performance MS in transmission mode, one needs to not only engineer phases and polarizations of locally transmitted waves but also suppress reflections simultaneously, which poses significant challenges in practical metaatom designs. Early designed transmissive meta‐atoms are typically multilayer metallic structures, involving Fabry‐Perot (FP) cavity resonances to enhance transmissions or impedance‐matched EM resonances (e.g., Huygens MSs) to suppress reflections [40, 41]. However, these meta‐atoms are still thick (∼λ/4), being unfavorable for integration‐optics applications. While dielectric MSs can avoid Ohmic losses, they are typically constructed by high aspect‐ratio dielectric pillars, which require enough thicknesses (∼λ/4) to ensure high transmission with wide phase coverage and polarization‐control capabilities [40, 42].

**FIGURE 1:**
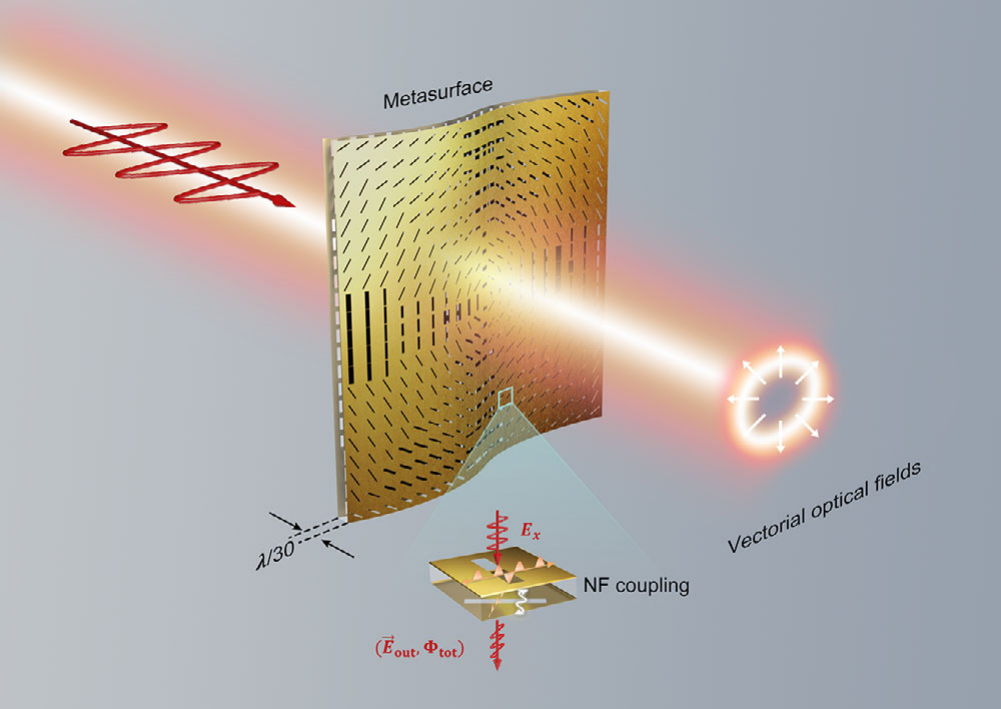
An extreme‐thickness metasurface to generate a vectoial THz beam in transmission mode, consisting of near‐field coupled air slits that can control the phases and polarizations of locally transmitted waves.

Here, we propose a new scheme to design MSs, exhibiting extreme thicknesses (∼λ/30), to generate and control vectorial beams in transmission mode, and experimentally verify the concept in the THz regime. Our basic metaatom consists of two coupled metallic screens, each drilled with an air slit with a specific orientation, which allows high transmission of THz wave with phase and polarization dictated by the geometries and orientations of two air slits (see Figure 1). Relying on near‐field (NF) coupling between two slit resonances, our meta‐atoms can be much thinner than a wavelength (∼λ/30). After experimentally characterizing the transmission properties of a series of basic meta‐atoms, we construct three THz metadevices and experimentally demonstrate that they can generate scalar Bessel beams with different polarizations and a cylindrically polarized Bessel beam, respectively, under illuminations of THz beams at 0.39 THz. Experimental results are in excellent agreement with full wave simulations.

## 2 Results and Discussion

### 2.1 Design and Characterization of MSs Components Based on LEM Theory

**FIGURE 2:**
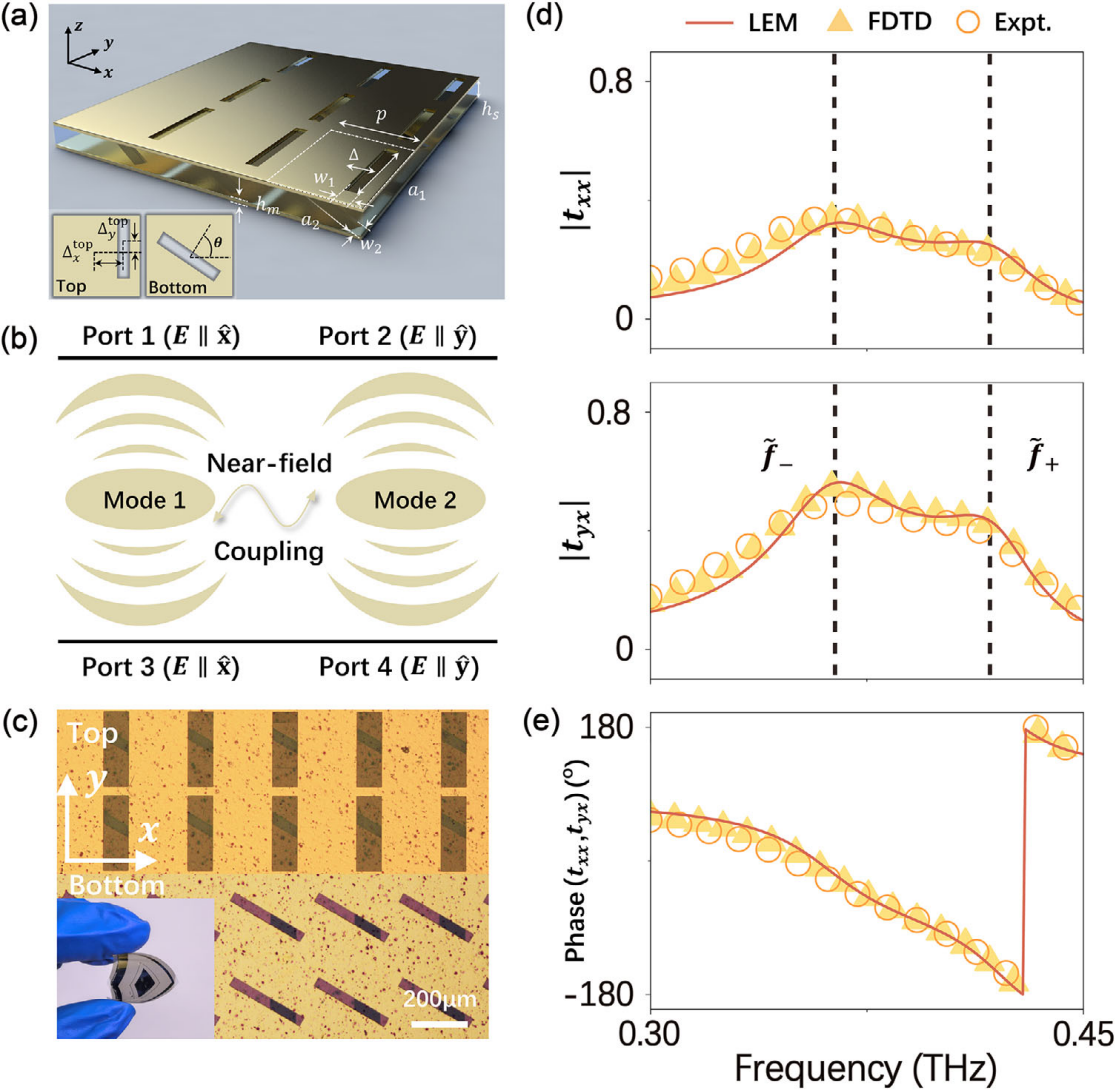
(a) Schematic of a bilayer periodic array of meta‐atoms containing two metallic screens with air slits exhibiting different sizes, orientations, and lateral offsets (see Inset). (b) A two‐mode, four‐port CMT model for the bilayer system. (c) Top‐ and bottom‐view pictures of a fabricated sample, with *p* = 328.6 µm, *a*_1_= 293.5 µm, *w*_1_= 86 µm, *a*_2_= 317 µm, *w*_2_= 26.5 µm, *h_s_* = 26 µm, *h_m_* = 0.15 µm, $\Delta_{x}^{\mathrm{top}}$ = 60 µm, and θ  =  60^○^. The spacer is a 26µm‐thick PET with permittivity ε_*r*_= 2.8 and loss tangent tanδ = 0.04. (d) Spectra of transmission amplitudes of the designed/fabricated bilayer system, obtained by LEM calculations (solid lines), FDTD simulations (triangles), and experimental measurements (circles). (e) This design employs LEM calculations, FDTD simulations, and experimental data to determine the corresponding transmission phase spectrum.

We analyzed the structures and corresponding functions of the meta‐atoms by near‐field coupling effects and leaky‐eigenmode (LEM) theory. Figure 2a depicts a typical metaatom consisting of two metallic screens separated by an extreme‐thickness dielectric spacer, with each screen perforated with an oriented air slit. Within the coupled mode theory (CMT) framework, a bilayer periodic array of such meta‐atoms can be modeled as a two‐mode, four‐port system (Figure 2b), with ports 1–2 (3–4) corresponding to the incident (transmitted) channels for ***x***‐ and ***y***‐polarized waves:

(1) $$\left\{\begin{array}{c} -i\omega \;a_{m}=-i\left(\omega_{m}-i\Gamma_{m}-i\Gamma_{m}^{a}\right)a_{m}+\sum_{n\neq m}\left(-it_{mn}+X_{mn}\right)a_{n}+\sum_{q}d_{qm}s_{q}^{+}\\ \;s_{q}^{-}=\sum_{p}s_{p}^{+}c_{qp}+\sum_{m}a_{m}d_{qm}\; \end{array}\right.$$

Here, *a_m_* (*m*  =  1, 2) are the modal amplitudes of the upper and lower slits, with ω_*m*_, Γ_*m*_, and $\Gamma_{m}^{a}$ denoting their eigenfrequencies, radiative losses, and absorption losses, respectively. The parameters *t_mn_* and *X_mn_* describe the NF and FF inter‐mode couplings, $s_{p}^{+}$ and $s_{q}^{-}\;\left(p,q=1,2,3,4\right)$ are the incoming and outgoing wave amplitudes at the external ports, *d_qm_* characterizes the mode–port coupling, and *c_qp_* represents the background scattering matrix.

All CMT parameters are analytically obtained using our recently developed LEM theory [43]. The LEM wavefunctions of the resonant modes, $\Psi_{m}^{\mathrm{LEM}}$, are obtained analytically or numerically and decomposed as $\;\Psi_{m}^{\mathrm{LEM}}=\Psi_{m}^{\mathrm{NF}}+\Psi_{m}^{\mathrm{FF}}$, and from which all CMT parameters are directly evaluated via field integrations (Note S1). Without retrieving CMT parameters by fitting with full‐wave simulations, our LEM theory can predict the optical responses of meta‐atoms with varying geometries, which are crucial for exploring the underlying physics and fast designing metadevices with tailored functionalities. By solving Equation (1), we can obtain an expression for transmission coefficient.

(2) $$t=\left(W_{+}{\tilde{d}}_{12}-W_{-}{\tilde{d}}_{11}\right)/W_{+}W_{-}$$

with $W_{\pm }=-i*\left(\omega -{\tilde{\omega }}_{\pm }\right)+{\tilde{\Gamma }}_{\pm }+{\tilde{\Gamma }}_{\pm }^{a}$. As shown in Equation (2), coherent interference between the two hybridized resonant modes ${\tilde{\omega }}_{-}$ and ${\tilde{\omega }}_{+}$ suppresses the reflection channel in the weak‐absorption regime, specifically when the absorption damping $\Gamma_{m}^{a}$ is nearly zero, leading to near‐unity transmission. The dual resonances further induce two π‐scale phase evolutions across the frequency domain, enabling continuous 2π coverage of the transmission phase while maintaining high transmittance. Here, ${\tilde{\omega }}_{\pm }$ denotes the frequency at which high transmission occurs after hybridization (Note S1).

We now employ the LEM theory to analyze the bilayer system as shown in Figure 2a. With all single‐resonance properties known (Note S1), we can analytically compute all CMT parameters including *t_mn_* and *X_mn_*, which critically depend on the relative configuration between two slits (e.g., the spacer thickness *h_s_*, inter‐slit lateral distance Δ, and the orientation angle θ of the bottom slit). Put the LEM‐computed parameters into Equation (1), we can predict the transmission/reflection spectra of coupled systems with different relative configurations from Equation (2). Solid lines in Figure 2d,e depict the LEM‐computed transmission amplitude/phase spectra of a particular bilayer system with top and bottom slits orientated along different directions, which are in excellent agreement with finite‐difference‐time‐domain (FDTD) simulations (triangles). Such quantitative agreement unambiguously demonstrates the predictive capabilities of our LEM theory. We fabricated the THz MS using a stripping process according to the design. To impart conformability, we employed a flexible polyethylene terephthalate (PET) layer (ε = 2.8; tanδ ≈  0.04) as the spacer layer (see Figure 2c for its top‐ and bottom‐view images and the entire flexible sample and experimentally characterize its transmission/reflection spectra using a THz time‐domain spectroscopy (TDS) system (Note S5). Measured spectra are shown as open circles in Figure 2d,e, which are in excellent agreement with both simulations and LEM predictions. More examples with different geometries are also analyzed (Notes S1 and S4).

We find from the calculated/measured spectra that waves transmitted through the meta‐atoms can be significantly modulated by altering the relative configuration of two air slits. First of all, we note that NF coupling *t_mn_* generates two hybridized modes at frequencies ${\tilde{f}}_{-}$ and ${\tilde{f}}_{+}$ (dash lines in Figure 2d), and thus the transmission phase varies continuously as frequency passes from ${\tilde{f}}_{-}$ to ${\tilde{f}}_{+}$. As *t_mn_* is dictated by evanescence‐wave coupling, the thinner the *h_s_*, the larger the *t_mn_*. Meanwhile, if *t_mn_* is too large, two hybridized modes are far separated in frequency, yielding a low‐transmission window within [${\tilde{f}}_{-}$, ${\tilde{f}}_{+}$] (Notes S1 and S2). An optimized *t_mn_* value is obtained by matching the frequency interval ${\tilde{f}}_{+}-{\tilde{f}}_{-}$ with the average bandwidths of two hybridized modes, which in our case corresponds to a spacer thickness *h_s_* =  26µm≈ λ_0_/30 with λ_0_ being the working wavelength. Such a device thickness is much thinner than all transmissive meta‐atoms realized previously [40, 42, 44, 45, 46]. In addition, we can achieve similar functions in the THz and even higher‐frequency regimes (e.g., the infrared regime) where metallic loss is relatively high, by utilizing dielectric metasurfaces (Note S9). Moreover, polarization state of transmitted wave can also be modulated. As shown in Figure 2d, while the incident polarization is along the *x* axis, the transmitted wave contains both *x*‐ and *y*‐polarized components indicating the polarization conversion.

We present phase diagrams to guide designing meta‐atoms exhibiting desired transmission phases and polarization‐conversion capabilities. In the thin‐slit limit, $\Psi_{m}^{\mathrm{NF}}$ is strictly polarized along the direction perpendicular to the *m*‐th slit, and thus the polarization of transmitted wave is solely dictated by the orientation angle of the bottom slit (Note S1). In practical designs, we fix the upper slits of all meta‐atoms to along the *y* direction and rotate the bottom slit by an angle θ. Thus, under illuminations of *x*‐polarized THz waves with amplitude *E*_in_, waves transmitted through these meta‐atoms can be generally written as

(3) $${\vec{E}}_{\mathrm{out}}=e^{i\Phi_{\mathrm{tot}}}\;\left(\cos\theta \hat{x}+\sin\theta \hat{y}\right)E_{\mathrm{in}\;}$$

**FIGURE 3:**
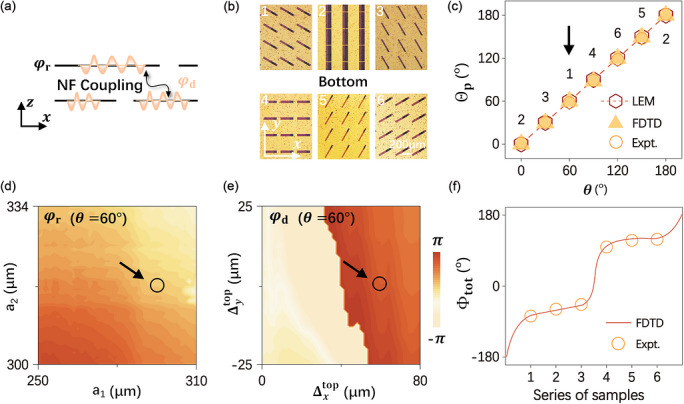
(a) Origins of the resonance‐induced phase φ_r_ and the detour phase φ_d_. (b) Bottom‐view pictures of six fabricated samples. (c) Polarization angle Θ_p_ of waves transmitted through meta‐atoms with different bottom‐slit orientation angle θ at 0.39 THz, obtained by LEM calculations, FDTD simulations and experimental measurements on six samples. (d) FDTD‐simulated φ_r_ for meta‐atoms with varying slit‐lengths *a*_1_ and *a*_2_ with other geometric parameters fixed as *a*_1_= 293.5 µm, *a*_2_= 317 µm, *w*_1_ = 86 µm, *w*_2_ = 26.5 µm, $\Delta_{x}^{\mathrm{top}}$ = 60 µm, θ = 60°. (e) FDTD‐simulated φ_d_ for meta‐atoms with varying offset distances $\Delta_{x}^{\mathrm{top}}$ and $\Delta_{y}^{\mathrm{top}}$, with other geometric parameters the same as in (d). (f) Measured Φ_tot_ of six fabricated samples, compared with FDTD simulations on a series of samples containing the six designed samples (Note S4). Open circles in (d,e) represent the designed sample 1.

Triangles in Figure 3c represent the polarization angles Θ_p_ of waves transmitted through a series of meta‐atoms with varying θ but with different slit width *w*, obtained by FDTD simulations at 0.39 THz. We find that all calculated results fall in the same Θ_p_ =  θ line, reinforcing our previous notation.

We now discuss how to tune the transmission phase Φ_tot_. At a fixed frequency, we can always tune Φ_tot_ through varying the lengths (*a*_1_,*a*_2_) of two slits, which in turn, shifts ${\tilde{f}}_{-}$ and ${\tilde{f}}_{+}$ accordingly (see Figure 2e). To illustrate such a mechanism, we depict in Figure 3d how the transmission phase governed by this mechanism (denoted as φ_r_) varies against *a*_1_ and *a*_2_, calculated by FDTD simulations at 0.39 THz for a series of meta‐atoms with θ  =  60°. We find that variation of φ_r_ is restricted to a range of π, as only the high‐transmission window [${\tilde{f}}_{-}$, ${\tilde{f}}_{+}$] is utilized (see Figure 2e). To achieve a full 2π phase modulation with high amplitudes, we introduce an additional mechanism through laterally shifting the top metal slit relative to the metaatom center. As illustrated in Figure 3a, in addition to the resonance phase φ_r_, a detour phase φ_d_ can be gained for wave transmitted through the metaatom, contributed by the propagation phase experienced by generated surface waves passing across the lateral distance between two slits. In bilayer metasurface systems, by leveraging the subwavelength nature of surface waves and combining the detour phase with the resonant phase, full 2π phase coverage can be achieved with high transmission efficiencies. This mechanism is generally applicable to other bilayer metasurfaces that support surface‐wave propagation and inter‐layer coupling (Note S12). Figure 3e depicts how φ_d_ varies against $\Delta_{x}^{\mathrm{top}}$ and $\Delta_{y}^{\mathrm{top}}$, obtained by FDTD simulations on a series of meta‐atoms with θ  =  60^○^. The total transmission phase Φ_tot_ = φ_r_  + φ_d_ can thus be modulated in a full 2π range, through adjusting both the slit sizes and the inter‐slit offset (indeed, both the detour phase alone and the combination of resonant and detour phases can achieve 2π phase coverage, but the combined approach offers higher transmission efficiency (Note S11).

Guided by these phase diagrams, we design and fabricate six samples (see Figure 3b for their bottom‐view images) with different geometric parameters ($\theta ,a_{1},a_{2},\Delta_{x}^{\mathrm{top}},\;\Delta_{y}^{\mathrm{top}}$) and experimentally characterize their transmission phases and polarization‐control capabilities at 0.39 THz. Measured Θ_p_ and Φ_tot_ are shown as open circles in Figure 3c,f (different Θ_p_ and Φ_tot_ correspond to different efficiencies (Note S13), respectively, which are in excellent agreement with both LEM and simulated results (Note S4). These experimental results confirm that we can design meta‐atoms exhibiting tailored transmission phases and polarization‐conversion capabilities, laying a solid foundation for constructing extreme‐thickness MSs to control vectorial THz beams in transmission configuration. Reflections of these meta‐atoms are generally weak at working frequency, indicating that their efficiencies are mainly restricted by Ohmic losses (Note S6).

### 2.2 Extreme‐Thickness Meta‐Device for Wavefront Reshaping

In the preceding section, the polarization and phase control capabilities of the meta‐atoms were successfully demonstrated. To further validate their regulatory capabilities, we employ the previously designed meta‐atoms to construct distinct terahertz devices capable of generating scalar Bessel beams with different linear polarization states and cylindrically polarized Bessel beams. Experimental verification confirms their generation capability, with each device achieving extreme‐thickness.

**FIGURE 4:**
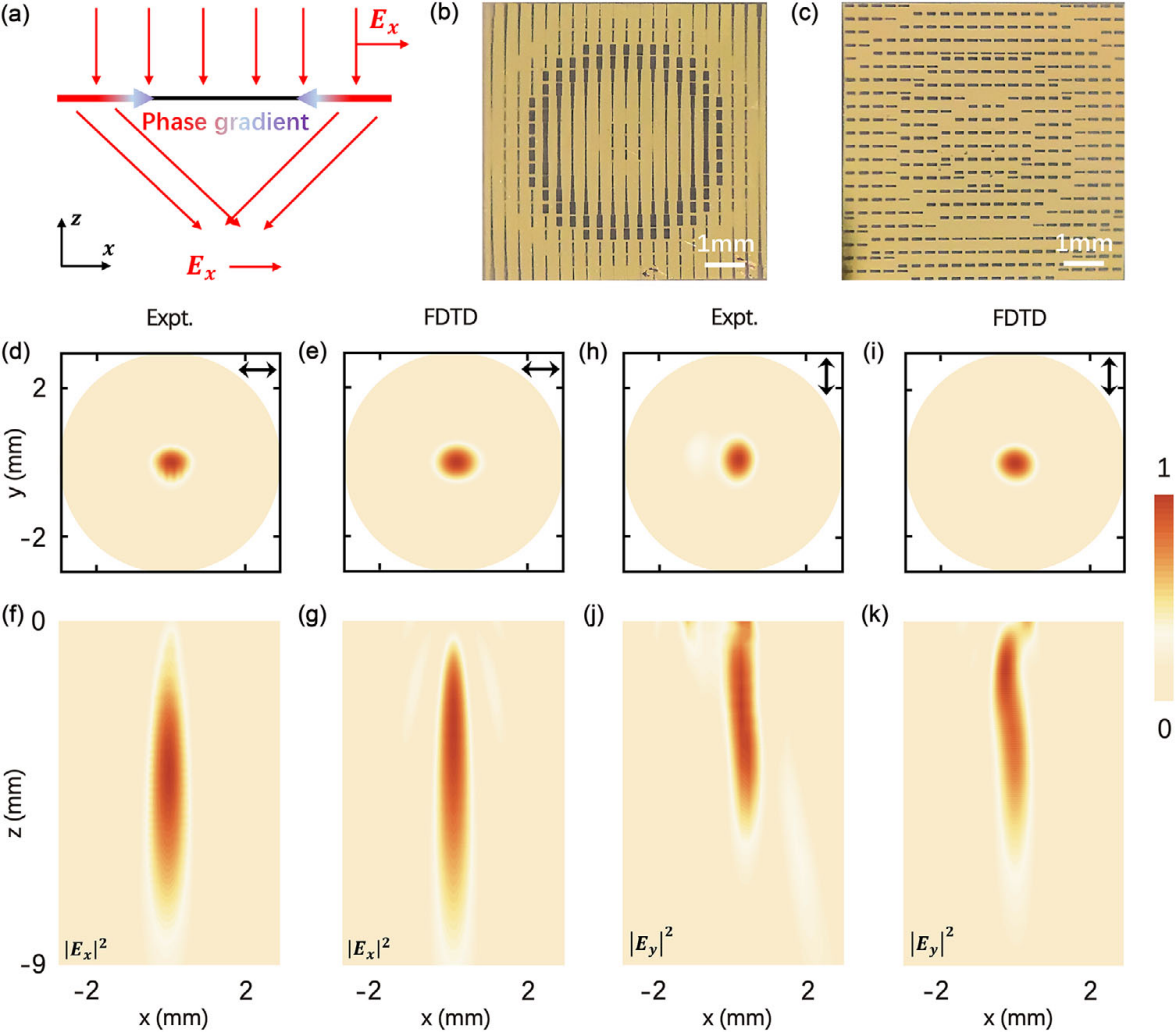
(a) Schematic of zeroth‐order Bessel beam generation based on MS. (b) Bottom‐view images of fabricated MS I whih θ  = 0°. (c) Bottom‐view images of fabricated MS II with θ  = 90°. (d) MS I measured the |*E_x_* |^2^ pattern on the *z*  =   − 4 *mm* plane at 0.39THz. (e) MS I simulated the |*E_x_* |^2^ mode on the *z*  =   − 4 *mm* plane at 0.39THz. (f) MS I measured the |*E_x_* |^2^ pattern on the *y*  =  0 *mm* plane at 0.39THz. (g) MS I simulated the |*E_x_* |^2^ mode on the *y*  =  0 *mm* plane at 0.39THz. (h) MS II measured the |*E_y_* |^2^ pattern on the *z*  =   − 4 *mm* plane at 0.39THz. (i) MS II simulated the |*E_y_* |^2^ pattern on the *z*  =   − 4 *mm* plane at 0.39THz. (j) MS II measured the |*E_y_* |^2^ pattern on the *y*  =  0 *mm* plane at 0.39THz. (k) MS II simulated the |*E_y_* |^2^ pattern on the *y*  =  0 *mm* plane at 0.39THz.

First, we employ the designed meta‐atoms to construct two MSs that can generate scalar zero‐order Bessel beams (see Figure 4a) exhibiting different linear polarizations. Fixing θ  = 0° and varying the geometric parameters ($a_{1},a_{2},\Delta_{x}^{\mathrm{top}},\;\Delta_{y}^{\mathrm{top}}$), we find a series of meta‐atoms that exhibit transmission phases satisfying

(4) $$\Phi =\Phi_{0}+k_{0}\sin\varphi *\sqrt{x^{2}+y^{2}}$$

at different local positions. Here Φ_0_ =  π/4, *k*_0_ is the wavevector of the incident light and φ = 16.56° (Note S7). We fabricate a sample according to the design, with its bottom‐view image depicted in Figure 4b. We find clearly that all slits are *y*‐orientated yet possessing different geometric parameters, adjusted to exhibit the required phases. In our experiment, we illuminate the sample by an *x*‐polarized beam at 0.39 THz and employ a NF probe to measure the |*E_x_* |^2^ distributions on two planes at the transmission side with *z*  =   − 4 *mm* and *y*  =  0 *mm*, respectively. Measured patterns are depicted in Figure 4d–f, respectively, in excellent agreement with corresponding simulation results (Figure 4e–g). These results clearly reveal the non‐diffractive nature of the generated Bessel beam, which exhibits a uniform polarization. To illustrate the powerfulness of our design scheme, we further design and fabricate another MS that can generate a cross‐polarized Bessel beam, based on meta‐atoms exhibiting θ  = 90° (see Figure 4c for the sample's bottom‐view image). It can be observed that, unlike the previous device, all slits are arranged along the x‐axis direction and possess distinct geometric parameters to generate the desired phase. Similarly, in this experiment, we illuminated the sample with an *x*‐polarized beam at 0.39 THz for testing. The difference lies in the fact that we ultimately obtained the |*E*_y_|^2^ distribution on two planes with *z*  =   − 4 *mm* and *y*  =  0 *mm*. The measurement results and simulation results are shown in Figure 4d–f and Figure 4e–g, respectively. The experimental and simulated results show high consistency. This demonstrates that our meta‐atoms can indeed achieve simultaneous control over phase and polarization. Different geometric parameters yield distinct transmission phases and linear polarizations, while extreme‐thickness terahertz devices formed from various meta‐atoms enable the manipulation of scalar fields.

**FIGURE 5:**
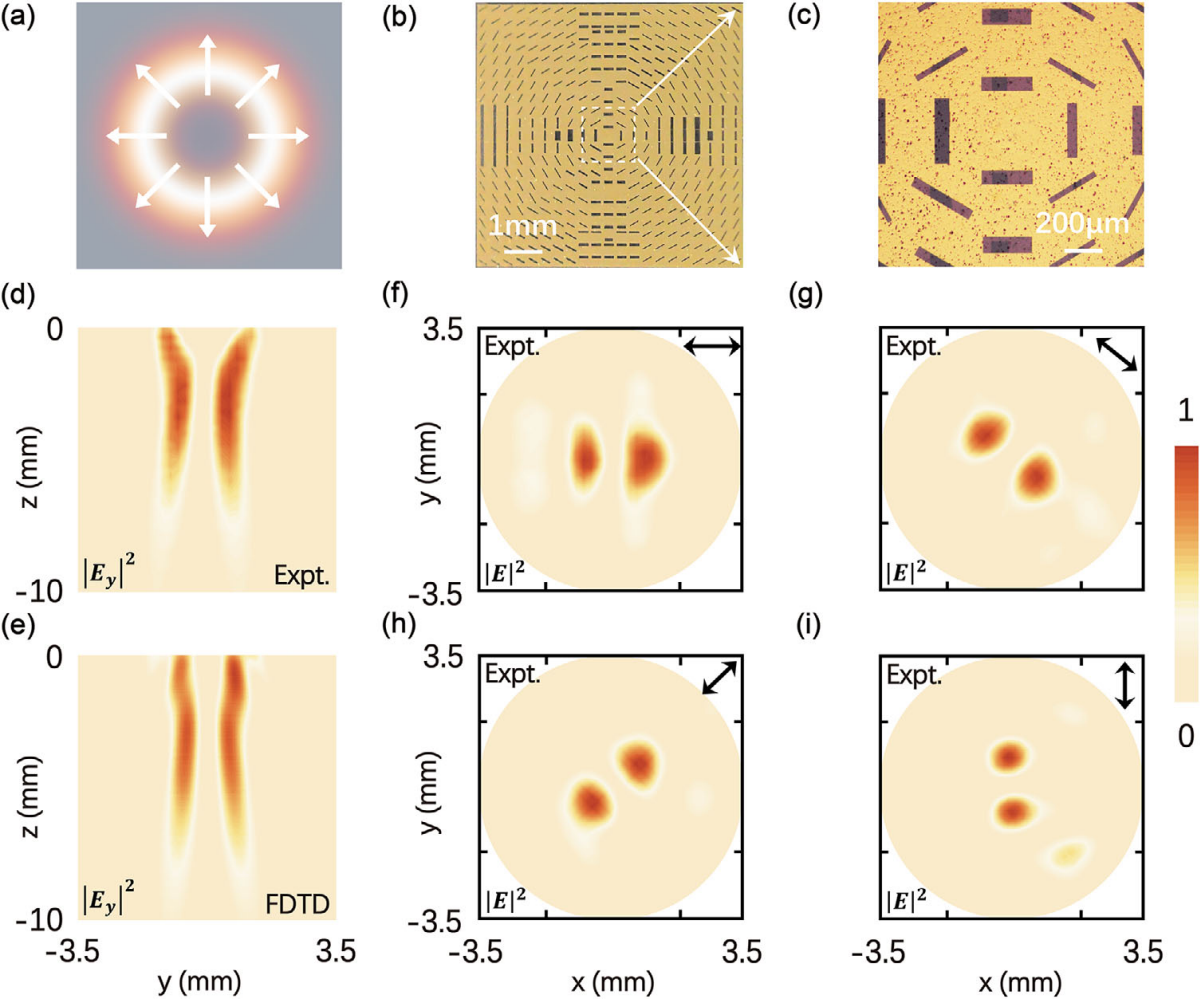
(a) Polarization distribution of the target vectorial Bessel beam on the *xy* plane. (b) Bottom‐view image of the fabricated MS. (c) Enlarged view of the central area of the bottom structure. (d) Measured |*E_y_* |^2^ patterns on the *x*  =  0 *mm* plane, as the MS is shined by an *x*‐polarized beam at 0.39 THz. (e) Simulated |*E_y_* |^2^ patterns on the *x*  =  0 *mm* plane at 0.39THz. (f) Measured ${|\vec{E}|}^{2}$ patterns on the *z*  =   − 4 *mm* plane at 0.39 THz with 0° linear polarization, i.e., x polarization. (g) Measured ${|\vec{E}|}^{2}$ patterns with ‐45° linear polarization. (h) Measured ${|\vec{E}|}^{2}$ patterns with 45° linear polarization. (i) Measured ${|\vec{E}|}^{2}$ patterns with 90° linear polarization, i.e., y polarization.

Encouraged by the excellent performance of the scalar extreme‐thickness metasurface, we further designed vectorial beam generation meta‐devices capable of producing radially polarized vector Bessel beams at 0.39 THz. The target beam is $E_{\mathrm{tar}}\left(r,t\right)=E_{0}\;J_{1}\left(k_{\rho }r\right)\;\left[\cos\alpha \;\hat{x}+\sin\alpha \;\hat{y}\right]\;e^{ik_{z}z}e^{-i\omega t},$ where $k_{\rho }^{2}+k_{z}^{2}=k_{0}^{2}$, *k*_ρ_ represents the lateral wavevector, α denotes the polar angle, and *J*_1_ is the first‐order Bessel function, as illustrated in Figure 5a. The desired phase distribution of this MS is also Equation (4), while the orientations of bottom‐layer slits should be adjusted to generate the desired local polarization states required by the target beam. According to the generic scheme established above, we design and fabricate a MS with its bottom‐view image shown in Figure 5b (see Figure 5c for a zoomed‐in picture). We experimentally characterize the functionality of this MS using the same NF mapping technology. Figure 5d depicts the measured |*E_y_* |^2^ distribution on the *yz* plane with *x*  =  0 *mm*, agreeing well with corresponding simulation results (Figure 5e). The zero‐field region in the beam center is a clear feature of a vector beam. To thoroughly investigate the vector properties of the generated beam, we simultaneously altered both the fixed direction of the samples and that of the NF probe, positioning them perpendicular to each other. We then mapped the light field distribution in the xy plane with *z*  =   − 4 *mm*. As shown in Figure 5f–i, measured patterns of E‐field polarized along different directions exhibit zeros along the angles perpendicular to the probing polarizations, unambiguously demonstrating that the generated Bessel beam is cylindrically polarized. The efficiencies of the Bessel‐beam generation devices are evaluated in Note S7. In addition to vector beams with all local polarizations being linear ones, generating other vector beams with more complex polarization patterns is also possible, based on meta‐atoms with bottom‐layer slits replaced by T‐shaped air slits (Note S8). We have also designed flexible samples under bending or stretching conditions, and our calculations demonstrate that they exhibit nice performances desired for flexible applications (Note S10). Therefore, our devices are thin while still effectively controlling the light field, paving the way for miniaturized transmissive terahertz components.

## 3 Conclusions

In summary, we propose a generic scheme to design transmissive MSs with extreme thicknesses (λ/30) to generate vectorial light beams, and experimentally verify the concept in the THz regime. The proposed metaatom consists of two vertically coupled air slits, which can control the phase and polarization state of transmitted wave upon varying the slit sizes, inter‐slits offsets and their orientations. We experimentally demonstrate three MSs, which can generate scalar Bessel beams with distinct linear polarizations and a cylindrically polarized Bessel beam, respectively, as shined by linearly‐polarized THz beams at 0.39 THz. Experimental results are in excellent agreement with full‐wave simulations. Relying on NF couplings between two slit resonances, our MSs are much thinner than previously realized MSs and possess flexible characteristics, which can find many applications in high‐performance on‐chip THz applications.

## Author Contributions

Yufei Song and Yixiang Xu contributed equally to this work. Yufei Song fabricated samples, conducted measurements, and carried out simulations; Yixiang Xu did the theoretical calculations, carried out simulations, and designed the samples; Yikai Xu conducted part of simulations; Yuanxi Liu provided assistance to Yufei Song in experiments. S.M. provided partial theoretical guidance. Q.H. provided technical supports for experiments and measurements. Z.W. and L.Z. conceived the idea and supervised the project. All authors contributed to the manuscript preparation, and have accepted responsibility for the entire content of this submitted manuscript and approved submission.

## Funding

This work was funded by the National KeyResearch and Development Program ofChina (No. 2022YFA1404700), the NationalNatural Science Foundation of China (Nos.12221004,12474306, 62192771,and12504436), Natural Science Foundation of Shanghai (Nos. 23xtcx00400 and 25ZR1402026).

## Conflicts of Interest

The authors declare no conflicts of interest.

## Supporting information




**Supporting File**: advs75898‐sup‐0001‐SuppMat.docx.

## Data Availability

The data that support the findings of this study are openly available from Science Data Bank (https://www.scidb.cn/s/6b6vU3).
