# Supplementary material for: Extreme‐Thickness Meta‐Membrane for Controlling Terahertz Vectorial Beams
Source: Adv Sci (Weinh). 2026 May 29;13(48):e75898. doi: 10.1002/advs.75898 (PMC13336914; doi:10.1002/advs.75898)
Supplement: Supplementary file 1 [file ADVS-13-e75898-s001.docx]

Supporting Information

Extreme-thickness Meta-membrane for Controlling Terahertz Vectorial Beams

*Yufei Song*^†^*, Yixiang Xu*^†^*, Yikai Xu, Yuanxi Liu, Shaojie Ma, Qiong He, Zhuo Wang^*^, Lei Zhou^*^*

**List of contents:**

**Section 1– LEM theory for our system**

**Section 2 – The impact of structural thickness on phase modulation**

**Section 3** **–** **Simulation of polarization perpendicular to the thin slit**

**Section 4 – Diagram of the six fabricated samples**

**Section 5 – Fabrication and Experimental test system**

**Section 6 – Reflection efficiency of meta atoms**

**Section 7 – Design strategy for Bessel beam**

**Section 8** **–** **Modulation of arbitrary polarization**

**Section 9** **– An all-dielectric metasurface system**

**Section 10 – Flexible material in bending and stretching conditions**

**Section 11 – Comparison of detour phase and resonant phase**

**Section 12 – More details about the detour phase**

**Section 13 – The effect of polarization and phase on the variation of the modulus of the transmission coefficient**

**Section 1** **–** **LEM theory for our system**

As for a generic coupled open system, we examine the scattering phenomena of a system composed of $m$ arbitrary resonators positioned at various locations within a host medium, subjected to specific external illumination. The region housing resonators is intricately linked to the external continuum through n ports, each characterized by distinct properties. And then we are required to solve the following Schrödinger-like equation:

$\hat{H}\Psi\left( \vec{r},\omega\right)=\omega\Psi\left( \vec{r},\omega\right)$	(S1.1)

where$\Psi\left( \vec{r},\omega\right)$ is the total wave function, and $\hat{H}=\hat{H}_{h}+\sum_{m} \hat{V}_{m}$ is the Hamiltonian of the whole system with $\hat{H}_{h}$ describing the host medium and the $\hat{V}_{m}$ potential contributed by the $m$th resonator.

Here, we define a set of wave functions$\left\{ \Psi_{m}^{\mathrm{LEM}}\left( \vec{r},\omega_{m} \right),m=1,\ldots,M \right\}$, which are the approximate solutions of the Hamiltonian $\hat{H}_{m}=\hat{H}_{h}+\hat{V}_{m}$, describing the subsystem containing only the $m$th resonator. And we can employ the following approach to obtain $\Psi_{m}^{\mathrm{LEM}}\left( \vec{r},\omega\right)$. We can solve $\hat{H}_{m}\Psi_{m}=\omega\Psi_{m}$ to obtain $\Psi_{m}$ either analytically or numerically, and subsequently derive the response spectrum of the system.

Next, we pinpoint the resonance frequency, denoted as $\omega_{m}$, of the $m$th resonator by locating the peak in the response spectrum. By selecting a background that characterizes the system at a frequency significantly distant from any resonances, we can derive the background wave function $\Psi_{B}$ through the illumination of the background medium with identical external light. And we can get the LEM wave function through $\Psi_{m}^{\mathrm{LEM}}=\Psi_{m}-\Psi_{B}$ for the $m$th resonator. According to leaky eigenmodes (LEM), $\Psi_{m}^{\mathrm{LEM}}\left( \vec{r},\omega_{m} \right)$ contains both NF and FF part:

$\Psi_{m}^{LEM}\left( \vec{r},\omega_{m} \right)=\Psi_{m}^{NF}\left( \vec{r},\omega_{m} \right)+\Psi_{m}^{FF}\left( \vec{r},\omega_{m} \right)$	(S1.2)

Where $\Psi_{m}^{\mathrm{NF}}$ and $\Psi_{m}^{\mathrm{FF}}$ represent the NF and FF parts of the wave function respectively. Generally speaking, for any given system with clearly defined external ports, we can consistently project $\Psi_{m}^{\mathrm{LEM}}$ onto the port modes on reference planes of all external ports. Then, we can construct $\Psi_{m}^{\mathrm{FF}}$ using these port modes, which are assumed to fill the entire space. With $\Psi_{m}^{\mathrm{FF}}$ determined, we can then obtain $\Psi_{m}^{\mathrm{NF}}$ numerically according to Equation S1.2.

Finally, we can construct the total wave function as

$\Psi\left( \vec{r},\omega\right)=\sum_{q} s_{q}^{+}\Psi_{B}^{q}+\sum_{n} a_{n}\left. |\Psi_{n}^{LEM} \right\rangle$	(S1.3)

Where $\left\{ a_{n} \right\}$ represents a set of unknown coefficients representing the strengths of fields scattered by different resonators under external illumination represented by $\left\{ s_{q}^{+} \right\}$ denoting the excitation amplitudes at different incoming ports. And $\Psi_{B}^{q}$ denotes the background wave function obtained when only the *q*th port is excited with unit amplitude. By substituting Equation S1.3 into Equation S1.1 and projecting both sides with $\left\langle\Psi_{n}^{\mathrm{NF}}| \right.$, then we can get the following equations to determine $\left\{ a_{n} \right\}$:

$-i\omega a_{m}=-i\left( \omega_{m}-i\Gamma_{m} \right)a_{m}+\sum_{n\neq m} \left( -it_{mn}+X_{mn} \right)a_{n}+\sum_{q} d_{qm}s_{q}^{+}$	(S1.4)

Next, we multiply both sides of $\Psi\left( \vec{r},\omega\right)$ defined in Equation S1.3 by each FF outgoing basis $\left\langle k_{q}^{-}| \right.$ (LSA), and then perform the field integrations at the reference planes of all ports. We finally can obtain the following equations:

$s_{q}^{-}=\sum_{p} s_{p}^{+}c_{qp}+\sum_{m} a_{m}d_{qm}$	(S1.5)

Which describes the strengths of scattered fields measured at different external ports.

Now, let us see our theory in photonic systems, which is described inhomogeneous permittivity function $\varepsilon(\vec{r},\omega)$ in which at each local point $\vec{r}$, the permittivity is $\varepsilon\left( \omega\right)=\varepsilon_{\infty}[1+\frac{\omega_{p}^{2}}{\omega_{0}^{2}-\omega^{2}+i\omega\Gamma_{e}}]$,where $\varepsilon_{\infty},\omega_{0},\omega_{p}$ and $\Gamma_{e}$ are all position- and frequency-independent parameters, describing the local properties of constituent materials. The governing equations (i.e., Maxwell’s equations in the frequency domain) can be formally rewritten as Equation S1.1, here the Hamiltonian is given by

$\hat{H}=\left( \begin{matrix} 0 & -\frac{i}{\mu}\nabla\times& 0 & 0 \\ \frac{i}{\varepsilon_{\infty}}\nabla\times& 0 & 0 & \frac{i}{\varepsilon_{\infty}} \\ 0 & 0 & 0 & i \\ 0 & i\omega_{p}^{2}\varepsilon_{\infty} & -i\omega_{0}^{2} & -i\Gamma_{e} \end{matrix} \right)$	(S1.6)

And the wave function is defined as $\Psi\left( \vec{r} \right)=\left( \vec{H} \vec{E} \vec{P} \vec{J} \right),$where $\vec{E}, \vec{H},$ and $\vec{P}$ denoting the electric, magnetic, and polarization fields, respectively, and $\vec{j}=\frac{d\vec{P}}{t}$ describing the polarization current.

According to the Hamiltonian form of photonic systems (Equation S1.6), we find that the potential operator contributed by the m-th scatter as shown in Equation S1.7.

$\hat{V}_{m}=\hat{H}_{m}-\hat{H}_{h}= \left( \begin{matrix} 0 & -i\left( \frac{1}{\mu_{m}}-\frac{1}{\mu_{h}} \right)\nabla\times& 0 & 0 \\ i\left( \frac{1}{\varepsilon_{\infty,m}}-\frac{1}{\varepsilon_{\infty,h}} \right)\nabla\times& 0 & 0 & -i\left( \frac{1}{\varepsilon_{\infty,m}}-\frac{1}{\varepsilon_{\infty,h}} \right) \\ 0 & 0 & 0 & 0 \\ 0 & i\left( \omega_{p,m}^{2}\varepsilon_{\infty,m}-\omega_{p,h}^{2}\varepsilon_{\infty,h} \right) & -i\left( \omega_{0,m}^{2}-\omega_{0,h}^{2} \right) & 0 \end{matrix} \right)$	(S1.7)

Where $\varepsilon_{\infty,m}$, $\mu_{m}$ and $\omega_{0,m}$ are position-dependent functions describing the properties of the m-th scatter, and $\varepsilon_{\infty,h}$, $\mu_{h}$, and $\omega_{0,h}$ describe the homogeneous host medium. We consider the lossless case (i.e., $\Gamma^{a}=0$), and consider a special case that scatters formed by plasmonic metals (Au or Cu) and the host medium is just air, then we have $\varepsilon_{\infty,m}=\varepsilon_{\infty,h}=\varepsilon_{0}$, $\mu_{m}=\mu_{h}=\mu_{0}$ and thus Equation S1.7 can be simplified as

$\hat{V}_{m}=\left( \begin{matrix} 0 & 0 & 0 & 0 \\ 0 & 0 & 0 & 0 \\ 0 & 0 & 0 & 0 \\ 0 & i\omega_{p,m}^{2}\varepsilon_{0} & 0 & 0 \end{matrix} \right)$	(S1.8)

The parameters in Equation S1.4and Equation S1.8 are clearly defined and can be computed using the provided integrals. These integrals offer a straightforward method to determine the values of the parameters, ensuring clarity and precision in their calculation.

$\left\{ \begin{aligned} \Gamma_{m}=i\left\langle\psi_{m}^{\mathrm{NF}} | \hat{V}_{m} | \psi_{m}^{\mathrm{FF}} \right\rangle_{V} \\ t_{mn}=\left\langle\psi_{m}^{\mathrm{NF}} | \hat{V}_{m} | \psi_{n}^{\mathrm{NF}} \right\rangle_{V} \\ X_{mn}=-i\left\langle\psi_{m}^{\mathrm{NF}} | \hat{V}_{m} | \psi_{n}^{\mathrm{FF}} \right\rangle_{V} \\ \kappa_{mq}=-i\left\langle\psi_{m}^{\mathrm{NF}} | \hat{V}_{m} | \Psi_{B}^{q} \right\rangle_{V} \\ c_{qp}=\left\langle k_{q}^{-} | \Psi_{B}^{p} \right\rangle_{S} \\ d_{qm}=\left\langle k_{q}^{-} | \psi_{m}^{\mathrm{FF}} \right\rangle_{S} \end{aligned} \right.$	(S1.9)

Where “*V*” and “*S*” denote whether the integrals are performed over the entire volume or at the reference plane of a port.

The following presents the wave function of the resonators.

**Figure S1:** (a) Wavefunction of resonator 1. (b) Wavefunction of resonator 2.

We first determine the properties of two original resonant modes ($\omega_{m}$, $\Gamma_{m}$, and $\Gamma_{m}^{a}$), by studying two model metasurfaces each containing one metal-film with slit array but with another replaced by a continuous metal screen. Under illuminations of THz waves with **E** fields perpendicular to the metal slit, we numerically obtain the reflection spectra (triangles) of two model metasurfaces (see Figs. S2a and S2b), from which the resonant frequencies $f_{1}$ and $f_{2}$ are clearly identified. We next obtain the LEM-wavefunctions $\Psi_{m}^{\mathrm{LEM}}$ of these modes, then employ the NF and FF wave functions to analytically compute $\Gamma_{m}$ and $\Gamma_{m}^{a}$, and finally put all single-resonator parameters into Equation (1) to calculate the reflection spectra of two model systems. Solid curves in Figs. S1a-d are the LEM-calculated spectra, which are in excellent agreement with numerical simulations and experimental results. Regarding the reflections in Figure S2a to Figure S2d, it was not possible to achieve zero-degree reflectance in the experiments, leading to the presence of an angle that results in a frequency shift, we have provided an explanation in Section V, and we also present a simulation of non-zero-degree reflectance by hexagon in Figure S2a to Figure S2d.

**Figure S2:** (a-d) Two individual resonant systems with their spectrum. The dashed lines $f_{1}$ and $f_{2}$ represent the original resonant frequencies of the two independent single-metal slit structures. The calculated results in the transmission coefficient modulus and phase spectra are obtained from theoretical calculations (solid line), FDTD simulation results (triangles and hexagon), and experimental results (circles). (e-f) The transmission coefficient modulus and phase spectra of the artificial microstructure in Figure 2a.

Figures S2e and S2f illustrate the coupling system with a $\theta=$90$^{\circ}$ of the bottom metal slit, which demonstrates that our theoretical framework is applicable not only to the $\theta=$60$^{\circ}$ discussed in the main text, but also to other angles (see Sec. IV in Supplementary Materials). And the specific structural parameter are $p$ = 328.6 μm, $a_{1}$= 253.5 μm, $w_{1}$= 58 μm, $a_{2}$= 257 μm, $w_{2}$= 48.5 μm, $h_{s}$= 26 μm, $h_{m}$= 0.1 μm, $\Delta_{x}^{\mathrm{top}}$= 90 μm, $\Delta_{y}^{\mathrm{top}}$= 0 μm, $\Delta_{x}^{\mathrm{bot}}$= 0 μm, $\Delta_{y}^{\mathrm{bot}}$= 100μm, $\theta=0^{\circ}$.

When the two original modes $f_{1}$ and $f_{2}$ are coupled together, the system exhibits two hybridized modes $\tilde{f}_{+}$ and $\tilde{f}_{-}$. Here, we show the process of diagonalization:

$\mathbf{M}\left( \begin{matrix} \omega_{1} & t \\ t & \omega_{2} \end{matrix} \right)\mathbf{M}^{\boldsymbol{-1}}\boldsymbol{=}\left( \begin{matrix} \tilde{\omega}_{+} & 0 \\ 0 & \tilde{\omega}_{-} \end{matrix} \right)$	(S1.10)

And $\mathbf{M}$ can be expressed by

$\mathbf{M}=\left( \begin{matrix} \frac{\Delta\omega+\sqrt{\Delta\omega^{2}+t^{2}}}{\sqrt{t^{2}+\left( \Delta\omega+\sqrt{\Delta\omega^{2}+t^{2}} \right)^{2}}} & \frac{t}{\sqrt{t^{2}+\left( \Delta\omega+\sqrt{\Delta\omega^{2}+t^{2}} \right)^{2}}} \\ \frac{\Delta\omega-\sqrt{\Delta\omega^{2}+t^{2}}}{\sqrt{t^{2}+\left( \Delta\omega-\sqrt{\Delta\omega^{2}+t^{2}} \right)^{2}}} & \frac{t}{\sqrt{t^{2}+\left( \Delta\omega-\sqrt{\Delta\omega^{2}+t^{2}} \right)^{2}}} \end{matrix} \right)$	(S1.11)

**Section 2 – The impact of structural thickness on phase modulation**

**Figure S3:** (a) $h_{s}=$ 10 μm. (b) $h_{s}=$ 34 μm. (c) $h_{s}=$ 80 μm. structural parameter are $p$ = 328.6 μm, $a_{1}$= 253.5 μm, $w_{1}$= 58 μm, $a_{2}$= 257 μm, $w_{2}$= 48.5 μm, $h_{m}$= 0.15 μm, $\Delta_{x}^{\mathrm{top}}$= 90 μm, $\Delta_{y}^{\mathrm{top}}$= 0 μm, $\Delta_{x}^{\mathrm{bot}}$= 0 μm, $\Delta_{y}^{\mathrm{bot}}$= 100 μm, $\theta=90^{\circ}$.

As shown in the figure above, we can observe that when the structural thickness is relatively small, the near-field coupling strength of the system is significantly high. This results in a considerable splitting of the two resonance peaks, leading to a lower valley value between them. However, this specific range is precisely where effective phase modulation occurs. Consequently, when phase control is applied within this range, the transmittance tends to be low, which adversely affects the efficiency during this process. However, when the structural thickness is relatively large, we can see that the two resonance peaks have merged into one. This is due to the excessively small near-field coupling strength of the system, which also results in a decrease in transmittance. Upon examining the phase, we find that the phase coverage area is also quite narrow. Therefore, structures that are too thick are not conducive to effective modulation of both amplitude and phase. Thus, when we select an appropriate thickness, such as 34 µm as shown in the figure, we can observe that the near-field coupling strength is well at this point. The two peaks are split, while the transmittance in the valley region between them is also maintained, covering a broader phase range. After continuous optimization, we ultimately chose a thickness of 26 µm for our study.

Nevertheless, we understand that such an optimized thickness sensitively depends on the bandwidths of two original peaks. To further reduce the optimize thickness (and thus enhance the inter-mode coupling), we need to increase the bandwidths of two original modes, which can be achieved by enlarging the widths of two slits on two metallic screens. In fact, this is a balance between the near-field coupling strength and bandwidth. The bandwidth can be adjusted by the width of the metal slits. Two important factors should be considered when designing the structure. First, the width of the slits cannot be too wide, as it is constrained by the geometric parameters of the entire structure. Therefore, in Fig. 4Sa, we optimized the thickness to 20 μm (red line) and compared the results of optimize the width of the metal slits (blue line). It can be seen that we can indeed balance the excessive impact of resonance peak splitting caused by excessive near-field coupling strength by adjusting the bandwidth. The second factor to consider is the absorption of the real system. As shown in Fig. S4b, the blue line represents the 20 μm structure after optimizing the bandwidth. It can be seen that the modulus of the transmission coefficient is significantly lower compared to our previous 26 μm structure. Therefore, although we can indeed optimize to a thinner thickness in ideal conditions with losses condition, considering the real system, a thinner thickness leads to stronger near-field coupling, which in turn enhances the absorption effect.

**Figure S4:** (a) Adjusting the slits width to optimize the bandwidth. Structural parameters are $w_{1}$= 58 μm, $w_{2}$= 48.5 μm and $w_{1}$= 78 μm, $w_{2}$= 68.5 μm. (b) Comparison of the modulus of the transmission coefficients for 26 μm and 20 μm.

**Section 3** **–** **Simulation of polarization perpendicular to the thin slit**

We employed a structure with a slit wider than all those presented in this paper for numerical simulations. By incidenting *x*-polarized electromagnetic waves onto this structure, we observed that the emitted electromagnetic waves contain only the component perpendicular to the slit, specifically the *x* component. Thus, we can ensure that the electromagnetic waves emitted from the other structures discussed in this paper are also polarized perpendicular to the bottom metal slit.

**Figure S5:** The transmission coefficient modulus of $t_{yx}$ and $t_{xx}$ in (a) and (b), respectively.

**Section 4** **–** **Diagram of the six fabricated samples**

We have presented the spectral line diagrams of the 0°, 30°,60°, 90°, 120°, and 150° series structures mentioned below.

**Figure S6:** (a) Diagram of $\theta$ in structure. (b) Sample pictures. The transmission coefficient modulus and its phase are shown in (c).

Figure S6a shows all the polarization angles $\theta$ we considered, with a total of six polarization angles selected to cover the full range of polarization. Additionally, we fabricated samples for each of these polarization angles, as shown in Figure S6b, and displayed the transmission spectra and phase characteristics of these structures and the solid line represents the theoretical calculation results, the triangles represent the FDTD simulation results, and the circles represent the experimental results. It can be observed that all three match very well. And we present the parameters of samples follow，for pure polarization conversion meta-atoms, it is necessary to further adjust the offset position of the lower slit to further adjust the amplitude and phase:

Table S1: Parameters of samples

| $\theta(^{\circ})$ | $a_{1} (\mu m)$ | $a_{2}(\mu m)$ | $w_{1}(\mu m)$ | $w_{2}(\mu m)$ | $\Delta_{x}^{\mathrm{top}}(\mu m)$ | $\Delta_{y}^{\mathrm{top}}(\mu m)$ |
| --- | --- | --- | --- | --- | --- | --- |
| 0 | 316 | 319.5 | 121 | 111.5 | 54 | 0 |
| 30 | 249 | 284 | 50 | 24 | 55 | 0 |
| 60 | 293.5 | 317 | 98 | 38.5 | 60 | 0 |
| 90 | 253.5 | 257 | 58 | 48.5 | 90 | 0 |
| 120 | 293.5 | 317 | 98 | 38.5 | 60 | 0 |
| 150 | 249 | 284 | 50 | 24 | 55 | 0 |

Keeping all the aforementioned $\theta$ constant, we can achieve a 2π phase coverage at 0.39 THz by varying the geometric parameters and the detour phase as demonstrated in Figure 3d and Figure 3e. This allows us to obtain the solid line in Figure 3f of the main text using FDTD.

**Section 5 – Fabrication and Experimental test system**

To achieve an ultra-thin metasurface with a degree of conformal capability, we employed 26μm optical polyethylene terephthalate (PET; $\varepsilon$= 2.8;$\tan\delta\approx$0.04) as the substrate. The fabrication of ultra-thin THz metasurfaces, with MIM-structured, was carried out with a four-step process, as shown in Figure S7a. First, the PET substrate was smoothly attached to the silicon wafer using PI tape. A layer of silicon (5 nm) was deposited as an adhesion layer on the upper surface of the PET substrate using a high-vacuum magnetron sputtering instrument (PVD75). Subsequently, a layer of photoresist (AZ5214) was spin-coated onto the upper surface. The photoresist layer was patterned using photolithography, which required high-intensity exposure. After that, to achieve a uniform gold film coating over the soft surface, a magnetron sputtering system (DE500) was employed to deposit an 150nm gold layer. In the fourth step, the photoresist was stripped using a chemical reagent, the undesired gold film was subsequently lifted off. Owing to the distinctive properties of the substrate, physical delamination was performed using a rubber-tipped dropper to scrape off the material, substituting the conventional ultrasonic stripping method. The processing of the second side could be accomplished by repeating the same four-step procedure; however, during its second step, alterations in the photolithographic parameters became necessary. This was owing to the pre-existing front-side metal structures and the thermal deformation of the PET substrate that occurred during deposition, which generally mandated an increased exposure dose.

**Figure S7:** (a) Experimental process flow chart, and (b) shows images of samples fabricated using different processes with and without an Si layer.

Compared to the conventional fabrication process, a pre-deposition silicon coating step was incorporated. This modification was necessitated by the suboptimal adhesion of the PET substrate, which was caused by the presence of crystalline point defects and a high degree of crystallinity. Before spin-coating the photoresist (AZ5214), we deposited a nanoscale silicon layer on the PET substrate to enhance its adhesion and compatibility with the photoresist. Accordingly, different processing steps were employed to investigate the influence of the nanoscale silicon layer on the final sample structure. As shown in Figure S6b, the gold film at the edges of the structures on the PET substrate without the silicon layer exhibited evident jagged detachment. In contrast, the structures on the silicon-coated PET substrate remained entirely intact.

**Figure S8:** (a) Far-field terahertz TDS system, transmission spectrum measurement method. (b) Far-field terahertz TDS system, reflection spectrum measurement method. (c) Near-field Terahertz TDS System.

The far-field spectral measurements were performed using a terahertz time-domain spectroscopy (THz-TDS) system. The system's emitter and receiver were both configured for *x*-polarization. The acquired time-domain signals were subsequently converted to the frequency domain via Fourier transformation. For the measurement of the $t_{xx}$, the sample was secured directly at the central point between the excitation and receiver ports, as show in Figure S8a. For the $t_{yx}$ measurement, the final spectrum could not be directly acquired and necessitated a conversion using a polarizer. A polarizer was placed at a 45-degree tilt on the left side of the sample stage (ensure the transmission direction of the polariser lies within the same quadrant as the sample's outgoing polarisation direction), as show in Figure S7a, The final output signal is a hybrid signal comprising $t_{xx}$ and $t_{yx}$. After removing the $t_{xx}$ signal, the $t_{yx}$ signal is obtained. For the measurement of far-field reflection spectra, as shown in Figure S8b, our experimental approach employs a small-angle incidence method, with an angle of approximately 23 degrees.

For near-field testing, we employed the THz-TDS near-field system. As shown in the Figure S8c, we secured the sample onto the sample holder, then performed a near-field scan using the system, ultimately obtaining the corresponding phase diagram.

**Figure S9:** Top-view optical images of samples.

Here, we present top-view optical images of all samples mentioned in the main text. Figure S9a to Figure S9f depict the samples at $\theta$ of 0°- 150° and Figure S8g to Figure S9i depict the samples at $\theta$ of 0°, 90°and for the vectorial Bessel beam, respectively.

**Figure S10:** (a) and (b) are E-field of samples in Figure 4e and Figure 4i by simulations, respectively .

We present a schematic diagram of the field distribution for another vertical polarization corresponding to the structures shown in Figures 4i and 4i of the main text. It is evident that the intensity is quite weak.

**Section** **6 –** **Reflection efficiency of meta atoms**

We experimentally tested the reflectance of meta atoms at each $\theta$ compared with simulations (open circles and solid lines, respectively).

**Figure S11:** (a)-(f) Sample of $\theta=0^{\circ}$, $30^{\circ}$, $60^{\circ}$, $90^{\circ}$, $120^{\circ}$ and $150^{\circ}$, respectively.

At the resonant frequency, we observe that the reflectance of the meta atoms is very low, indicating that our designed system exhibits high transmittance properties.

**Section 7 – Design strategy for Bessel beam**

**Figure S12:** Schematic of phase gradient metasurface.

As shown in the figure, it illustrates the oblique angle $\varphi$，when an electromagnetic wave is normally incident on a phase-gradient metasurface. According to the principle of wavevector matching, we know that the horizontal wavevector at the incident interface must be equal to the horizontal wavevector at the exit interface. From this, we can derive the following equation.

$0+\xi=k_{0}sin\varphi$	(S1.12)

Here, represents the wavevector in vacuum, and we set the operating frequency to 0.39 THz. $\xi$ is the phase gradient of the metasurface we designed, with $\xi=\frac{d\phi}{dx}=\frac{-2\pi}{8*d}=-0.285k_{0}$. Therefore, we can finally calculate the exit angle $\varphi$ to be 16.56°. The simulated efficiency of the 90$^{\circ}$ and 0$^{\circ}$ scalar Bessel beam generator, disregarding substrate absorption, is 70.81％ and 40.13%; when accounting for absorption, the simulated efficiency is 57.22％ and 26.23%.

As mentioned in the main text, we divided the metasurface into 12 regions, following the principle of phase gradient, and populated them with artificial atoms of different polarizations we designed in above text, as shown in the figure below.

**Figure S13:** Schematic of Vectorial Bessel beam. Arrows of different colors represent different polarizations in the *xoy* plane.

In this way, when the electromagnetic wave illuminates the metasurface, the outgoing electromagnetic wave is controlled both by the phase, forming a tightly focused beam, and by the polarization, ultimately becoming a vector Bessel beam. The simulated efficiency of the vector Bessel beam generator, disregarding substrate absorption, is 64.10%; when accounting for absorption, the simulated efficiency is 31.17%.

As shown in Fig. S14, taking the 0$^{\circ}$ scalar Bessel metasurface as an example, we first direct the incident light along the -z direction into a region of air that is the same size as the metasurface, collecting the electric field distribution and integrating it to obtain the energy of the incident field. Similarly, we replace the air with the metasurface and collect the energy of the outgoing light beam in this dashed plane. Finally, we divide the outgoing energy by the incident energy to obtain the efficiency.

**Figure S14.** Electric field distribution when the incident light is illuminated on the air (a) and the metasurface (b).

In the table below, we present the efficiency of three Bessel beam devices at the operating frequency and near the operating frequency. It can be seen that the devices we designed also exhibit a certain degree of frequency dispersion.

Table S2: The efficiency of three Bessel beam devices

| Frequency (THz) Devices | 0.37 | 0.38 | 0.39 | 0.4 | 0.41 |
| --- | --- | --- | --- | --- | --- |
| 90$^{\circ}$ Bessel beam | 55% | 55% | 57% | 54% | 53% |
| $0^{\circ}$ Bessel beam | 23% | 25% | 26% | 26% | 24% |
| Vectorial Bessel beam | 30% | 31% | 31% | 30% | 30% |

**Section 8–** **Modulation of** **arbitrary polarization**

Our article mainly introduces the case where the emitted polarization is any linear polarization, and, combined with different linear polarization structures, we successfully designed a vector light field device. In fact, by modifying the underlying structure, it is also possible to convert a single linear polarization into any elliptical or circular polarization. We still take 0.39 THz as the operating frequency point and calculated a series of polarization conversion cases at this frequency (excluding absorption). Here, we show three different polarizations and their corresponding structures. As shown in Figure S14a, we adopted the same upper structure, only changing the parameters of the lower structure. For the upper structure, $a_{1}$= 253.5μm, $w_{1}$= 58μm, $\Delta_{x}^{\mathrm{top}}$ = 90μm, $\Delta_{y}^{\mathrm{top}}$= 0. For the bottom structure of the first polarization, $a_{2}$= 257μm, $w_{2}$ = 48.5μm, $\Delta_{y}^{\mathrm{bot}}$ = 100μm, $a_{3}$= 190μm, $w_{3}$ = 70μm, $\Delta_{x'}^{\mathrm{bot}}$ = 15, $\Delta_{y'}^{\mathrm{bot}}$ = -50μm. For the bottom structure of the second polarization,$a_{2}$= 257μm, $w_{2}$ = 48.5μm, $\Delta_{y}^{\mathrm{bot}}$ = 100μm, $a_{3}$= 220μm, $w_{3}$ = 70μm, $\Delta_{x'}^{\mathrm{bot}}$ = 15, $\Delta_{y'}^{\mathrm{bot}}$ = -45μm. For the bottom structure of the third polarization,$a_{2}$= 257μm, $w_{2}$ = 48.5μm, $\Delta_{y}^{\mathrm{bot}}$ = 100μm, $a_{3}$= 220μm, $w_{3}$ = 70μm, $\Delta_{x'}^{\mathrm{bot}}$ = 15, $\Delta_{y'}^{\mathrm{bot}}$ = -40μm. As shown in Figure S15, The spectral diagram corresponding to the second circularly polarized structure can be observed. Provided the underlying structure is appropriately designed to provide corresponding exits for both modes, distinct polarized outputs can ultimately be generated.

**Figure S15:** (a) Three different structural parameters and their corresponding polarization states, and (b) shows Circular polarization and the corresponding spectral lines

**Section 9** **– An all-dielectric metasurface system**

To broaden the practicality of the system, we replaced the metal structure with LTCC-C type ceramic ($\varepsilon_{r}=20)$ as suggested by the reviewer, designing an all-dielectric metasurface system.

**Figure S16.** Transmission spectrum of the all-dielectric metasurface. Structural parameter are $p$ = 328.6 μm, $a_{1}$= 253.5 μm, $w_{1}$= 58 μm, $a_{2}$= 257 μm, $w_{2}$= 48.5 μm, $h_{m}$= 60 μm,$h_{s}=$ 26 μm. $\Delta_{x}^{\mathrm{top}}$= 90 μm, $\Delta_{y}^{\mathrm{top}}$= 0 μm, $\Delta_{x}^{\mathrm{bot}}$= 0 μm, $\Delta_{y}^{\mathrm{bot}}$= 100 μm, $\theta=90^{\circ}$.

It can be seen that when using all-dielectric metasurfaces, the system still exhibits good transmission performance as shown in Fig. S15. And its thickness is 6/30 of the operating wavelength.

For the MIM metal structure, we scaled down the metasurface and slightly adjusted the parameters. Figure S17a shows the transmission coefficient magnitudes of the $\theta=90^{\circ}$ meta-atoms in the infrared bands. The results indicate that metal losses increase at higher frequencies, leading to reduced transmission efficiency. To mitigate this, we can replace gold by silver and substitute the intermediate dielectric layer by SiO_2_ to reduce absorption, on the expenses of losing flexibility. Simulations are performed on the structure with $\theta=90^{\circ}$ with a dielectric layer thickness of $0.6 \mu m$ and a silver film thickness of $30 nm$. The obtained results (Fig. S17b) show improved polarization conversion efficiency.

Moreover, replacing the metal layers with dielectric layers (top and bottom LTCC-C ceramic ($\varepsilon_{r}=20$) $1 \mu m$, middle SiO_2_ $0.43 \mu m$, with small rectangular holes in the top and bottom layers) significantly enhances the polarization conversion efficiency of $\theta=90^{\circ}$meta-atoms in the high-frequency range (Fig. S17c), indicating that dielectric structures are more suitable for high-frequency applications. The feasible fabrication process is as follows: deposit a SiO_2_ film on an LTCC-C substrate, laminate an LTCC-C layer to form an initial three-layer structure, etch the dielectric layer to the desired thickness, and expose/etch the front- and back-side structures sequentially. The thin substrate poses challenges for achieving the desired thickness, which can be addressed by laser etching or chemical solutions (e.g., 19 wt% NaOH) to reduce the substrate to the target thickness.

**Figure S17.** Simulation results of the transmission coefficient magnitude for the bilayer metallic structure in the infrared (a), for the silver bilayer metallic structure in the infrared (b), and for the dielectric metasurface in the infrared (c).

**Section 10** **– Flexible material in bending and stretching conditions**

We performed full-wave simulations using a metasurface that ultimately generates a vector Bessel beam as an example.We also define the light source as extending from the negative to the positive direction along the z-axis.

First, regarding the bending issue, as shown in Fig. S18a, we wrap the metasurfaces around a cylinder, using the total length of the metasurfaces as the arc length and the corresponding central angle as α. The corresponding YOZ cross-sections are shown in Figures S18b, c, and d. It can be seen that the metasurface continues to maintain good performance even when subjected to small-angle bending. For a bending central angle of 30°, the vector Bessel beam becomes slightly shorter, but the metasurface is still capable of performing its intended function.

**Figure S18.** Effect of bending on metasurface performance. (a) Schematic of bending methods and parameters. (b–d) Full-wave simulation results for α = 5°, 15°, and 30°, respectively.

For tensile problems, the amount of elongation and changes in thickness are primarily related to the applied stress $\sigma$, the Young's modulus $E$ of PET, and the Poisson's ratio $\nu$. The Young's modulus of the PET film used in the study is 2.49 GPa. The Poisson’s ratio of the PET film is 0.38. With these specific parameters, we can calculate the change in the edge length of the metasurface after stretching:

$\Delta L=\frac{\sigma}{E}\times L_{0}$	(S10.1)

Where $L_{0}$ is the initial length. The change in the thickness of the metasurface after stretching can also be calculated as follows:

$\Delta t=-\nu\times\frac{\sigma}{E}\times t_{0}$	(S10.2)

Where $t_{0}$ is the initial thickness. Within its elastic range, PET can generally withstand stresses of around 100 MPa; when the stress exceeds 200 MPa, PET will fracture. Here, we select three different stress levels: 50 MPa, 100 MPa, and 300 MPa (the latter two being extreme cases), and plot the corresponding Fig. S19b, c and d values for the YOZ cross-sections from full-wave simulations. It can be observed that even when subjected to extreme stress, the metasurface is capable of generating high-quality vector Bessel beams, indicating its robustness to tensile stress.

**Figure S19.** The Effect of stretching on metasurface performance. (a) Schematic of the stretching method. (b–d) Full-wave simulation results for σ = 50, 100, and 300 MPa, respectively.

**Section 11** **– Comparison of detour phase and resonant phase**

Both the detour phase alone and the combination of resonant and detour phases can achieve 2π phase coverage, but the combined approach offers higher transmission efficiency. The reason for combining these two mechanisms lies in the relationship between phase control and transmission efficiency. When tuning only the detour phase or only the resonant phase, although 2π coverage can be achieved, the corresponding phase range often does not fall within the high-transmission-efficiency region. This is because both phase contributions jointly affect the coupling strength between modes, which directly determines the transmission amplitude at the operating frequency. By adjusting both resonant and detour phases simultaneously, the desired 2π phase range can be positioned within the high-transmission-efficiency region. As shown in Fig. S20, the modulus of the transmission coefficient obtained using the combined phase-control approach is significantly higher than that achieved by tuning the detour phase alone.

**Figure S20.** Comparison of the modulus of the transmission coefficient between the combination of resonant and detour phase and the standalone detour phase.

**Section 12** **– More details about the detour phase**

Surface waves are inherently subwavelength, allowing significant phase accumulation even with subwavelength displacements of the meta-atoms. Based on this property, the detour phase introduces an additional optical path relative to the incident wave, producing an extra phase. In our bilayer metasurface system, the contribution of the upper metallic slit offset $\Delta_{x}^{top}$ relative to the center to the phase is illustrated in Fig. S21. Individually, tuning either the detour phase or the resonant phase can achieve 2π phase coverage, but the range may not align with high-transmission-efficiency regions; by combining both, the desired 2π phase range can be positioned within the high-efficiency region, balancing phase coverage and transmission efficiency. This mechanism is therefore most effective in structures supporting surface wave propagation and coupling system. Regarding its generality, previous studies ([*Adv. Funct. Mater. 2020, 1910610*], [*Optics & Laser Technology 181 (2025) 111645*], etc.) have demonstrated that other types of bilayer metasurface systems can also employ this approach for effective phase control.

**Figure S21.** The phase contributed by the additional optical path $\Delta_{x}^{top}$ that causes the detour phase.

**Section 13** **– The effect of polarization and phase on the variation of the modulus of the transmission coefficient**

To examine the effect of the slit rotation angle and geometric dimensions on transmission, we varied these two parameters. Since the outgoing phase is determined by the slit geometry and the output polarization corresponds directly to the slit rotation angle, we constructed a two-dimensional diagram in which the outgoing phase and polarization state span the two axes, and the transmission amplitude is represented by a color scale (Fig. S22). This diagram clearly shows that variations in either the polarization or the phase lead to corresponding changes in the transmission coefficient. Furthermore, because the overall metasurface device is composed of unit cells generating different polarizations and phases, fluctuations in the transmittance of individual units will inevitably impact the total device efficiency.

**Figure S22.** A two-dimensional phase diagram of the variation in the modulus of the transmission coefficient when the polarization and phase change.
